# Psychological Factors Associated with Phantom Limb Pain: A Review of Recent Findings

**DOI:** 10.1155/2018/5080123

**Published:** 2018-06-21

**Authors:** Xaver Fuchs, Herta Flor, Robin Bekrater-Bodmann

**Affiliations:** ^1^Biopsychology and Cognitive Neuroscience, Faculty of Psychology and Sports Science, Bielefeld University, Bielefeld, Germany; ^2^Department of Cognitive and Clinical Neuroscience, Central Institute of Mental Health, Medical Faculty Mannheim, Heidelberg University, Mannheim, Germany; ^3^Center for Neuroplasticity and Pain (CNAP), SMI®, Department of Health Science and Technology, Aalborg University, Aalborg, Denmark

## Abstract

Phantom limb pain (PLP) is a common phenomenon occurring after the amputation of a limb and can be accompanied by serious suffering. Psychological factors have been shown to play an important role in other types of chronic pain, where they are pivotal in the acquisition and maintenance of pain symptoms. For PLP, however, the interaction between pain and psychological variables is less well documented. In this review, we summarize research on the role of emotional, motivational, cognitive, and perceptual factors in PLP. The reported findings indicate that emotional factors modulate PLP but might be less important compared to other types of chronic pain. Additional factors such as the amount of disability and adjustment to the amputation appear to also play a role. Bidirectional relationships between stress and PLP have been shown quite consistently, and the potential of stress and tension reduction in PLP treatment could be further exploited. Little is known about the role of cognitive variables such as attention or expectation. Catastrophizing seems to aggravate PLP and could be targeted in treatment. Body perception is altered in PLP and poses a potential target for novel mechanistic treatments. More research on psychological factors and their interactions in PLP is needed.

## 1. Introduction

The amputation of a limb represents a serious disruption of body integrity and is associated with a number of negative consequences, particularly disability and postamputation pain. Although severe chronic residual limb pain is rare (occurring in less than 10% of the amputees), the majority of amputees (above two-thirds) report at least mild residual limb pain [1]. As a consequence of changes in the posture and strain of the remaining body parts, secondary pain is reported by a high proportion of the amputees, with high rates (more than 20% each) of pain in the remaining limbs, the shoulders, and the neck or the upper back [2, 3]. Almost all amputees also report about ongoing awareness of a phantom limb, with 60–80% complaining about phantom limb pain (PLP), that is, a painful sensation perceived in the amputated limb and thus located outside the physical borders of the body [4–6]. PLP usually begins early after an amputation (e.g., [7]), and in most amputees, PLP becomes chronic with a large variability in intensity, frequency, and quality [6, 8]. PLP leads to personal suffering [9], reduced quality of life [10], and impairs sleep [6]. PLP is still a challenge for pain research and treatment.

Neuroplastic changes occurring in the brain after an amputation have been a major focus in PLP research for the last two decades (for reviews, see [11, 12]). In contrast, less research has focused on psychological factors, and although they play an important role in other types of chronic pain, for example, chronic musculoskeletal pain (e.g., [13–15]), their role in PLP remains less well studied and less well understood (e.g., [16]). Furthermore, review articles directly addressing psychological factors in PLP are rare. Sherman et al. [17] and Hill [18] reviewed and critically discussed evidence from earlier studies and showed that PLP cannot be related to unresolved grief due to limb loss, denial, psychosomatic manifestations, pathological misinterpretation of somatic sensations, or personality disorders (e.g., [19–21]). The proposed relationship between personality factors and PLP could not be substantiated ([17]; for a discussion, see [18]).

Since the reviews cited above [17, 18] have been published two and three decades ago, respectively, the present work focuses and summarizes the research on the role of psychological variables from the late 90s to the present. We further focus on emotional factors, motivational aspects such as stress and stress coping, cognitive factors, and the role of body perception in the development and maintenance of chronic PLP and its potential for treatment approaches.

## 2. Emotional Factors

Much research on emotional processing in chronic pain has focused on comorbid anxiety and depression, which have a high prevalence in chronic pain (cf. [22–24]). For example, a health survey in a representative German sample [25] found an interview-based [26] prevalence of 8.1% for chronic pain disorder. Compared to the healthy population, the 12-month prevalence for anxiety disorders was significantly elevated in both male (33% versus 7%, odds ratio = 5.65) and female (37% versus 20%, odds ratio = 2.69) chronic pain patients. Mood disorders were also significantly more prevalent in chronic pain patients compared to pain-free participants (men: 30% versus 7%, odds ratio = 5.48; women: 30% versus 15%, odds ratio = 2.69). In line with the role of anxiety and mood disturbances (especially depression) in chronic pain, similar relationships can also be expected for chronic PLP. In the following sections, we will summarize findings on the role of depression and anxiety in PLP and will show that it is important to take specific characteristics of the sample into account, especially whether the sample consisted of amputees in early or later stages after amputation and whether concomitant pain was present.

### 2.1. Depression and Anxiety in Early-Stage Amputees

Especially in early stages after amputation, comorbidity rates of mental disorders in amputees can be related to factors other than PLP. Factors like chronic diseases leading to the amputation, traumatization, secondary pain, disability caused by the amputation, and adaptation to the new situation can give rise to anxiety and depression independently of PLP. The modulation of pain by emotional factors may therefore be different in early and late stage amputees. For example, Shukla et al. [27] reported high rates of depression (about 50%) and anxiety (above 35%) in amputees in the postoperative phase, regardless of PLP. In contrast, in samples that were more heterogeneous in age, time since amputation, or the cause of amputation, prevalence rates for depression and anxiety were lower than the rates reported by Shukla et al. [27] for recently amputated participants. For example, a prevalence of 19% was reported for depressive symptoms [28] and of 24% for both depression and anxiety symptoms [29]. In these studies, the association of depression and anxiety with PLP (which is most prevalent shortly after amputation [1]) has not been specifically investigated. Consequently, recent amputations should be seen as a special case, and in fact, there is evidence that depression in the postamputation phase is more strongly related to concerns about disability than PLP [28, 30, 31]. However, a different relationship has been observed for preamputation anxiety. Raichle et al. [32] investigated the relationship between overall anxiety levels prior to lower limb amputation and PLP and found that they were positively related particularly with PLP intensity (up to five days after amputation), even when postoperative analgesic medication was controlled for. The analysis of the interplay between emotional states and PLP can be better determined in later stages, when disability and adaptation to the new situation are no longer dominating topics. However, even in later stages, PLP is frequently related to disability and it is important to consider both disability- and pain-related issues.

### 2.2. Depression in Later Stages after Amputation

As already stated, symptoms of depression are common in the acute phase after an amputation. However, several studies showed that the rates of depressive symptoms decline during the following years. Horgan and MacLachlan [33] published a review on psychosocial adjustments following lower limb amputations and concluded that depression in the first years after amputation is most strongly associated with disability and that, by two years after amputation, depression rates have dropped to a level comparable to those of healthy people. However, even at later stages, it is important to distinguish between disability, somatic symptoms, pain, and depression. Whyte and Niven [31] used the Beck Depression Inventory (BDI [34]) to assess depressive symptoms in a sample of amputees with PLP in the chronic stage. They observed a significant positive correlation of PLP and BDI scores; however, they found that this correlation was mainly driven by PLP being correlated with items of the BDI that assess performance or somatic symptoms that are often seen in chronic pain. The problem that the BDI (and possibly other depression scales as well) tends to overestimate depression in samples with physical diseases and chronic pain has been pointed out before [35]. This highlights that the diagnostic instrument needs to be taken into account in the interpretation of studies on amputees and that psychopathology might be overestimated due to somatic symptoms associated with amputations. Desmond and MacLachlan [36] used the Hospital Anxiety and Depression Scale (HADS [37]) to screen for anxiety and depression and the Impact of Event Scale [38] to assess the severity of posttraumatic stress. They tested a sample comprising exclusively older male amputees (*N*=582) with long-standing traumatic amputation (at least 10 years). Importantly, the HADS specifically excludes items concerning intrusions or physical symptoms of depression to avoid confounding when assessing groups with somatic disorders. In this homogeneous sample, the authors found elevated scores for depression, anxiety, and posttraumatic stress. In the amputee sample, values potentially indicating clinically relevant depression were observed three times more often than expected for the normal population. This study also found differential associations between psychological distress and postamputation pain: amputees showed higher values in depressive symptoms, avoidance, and intrusions if they suffered from either residual limb pain or residual limb pain *and* PLP but not if they suffered from PLP alone, indicating that chronic PLP might be different from other postamputation pain in terms of psychological distress. However, it is not entirely clear which factors caused the differences between the subgroups. The authors assumed that residual limb pain more strongly conflicts with prosthesis use so that their results might have been associated with activity restriction-induced negative affect [39]. Desmond and MacLachlan's [36] results suggest that PLP and depression are—if at all—only weakly associated if residual limb pain is accounted for. However, their sample consisted of former military service members who were mostly male, at younger age when serving, and suffered from traumatic limb loss in the line of duty with no preceding pain or diseases, and thus, these findings should not be generalized to other populations. Another study [40] reported the results of a survey that used a stratified sample of 914 amputees, which might provide more general norms. To assess depressive symptoms, the authors used the Center for Epidemiological Studies Depression Scale [41], which is also better suited for assessing depression in populations with chronic health problems. Pain bothersomeness was assessed individually for PLP, residual limb pain, and back pain. There was a significant correlation between pain bothersomeness and depressive symptoms within each of the groups. Depressive symptoms were almost three times more likely to be present in subjects who were extremely bothered by PLP than in subjects reporting not to be bothered. However, this association was even stronger in residual limb pain and back pain. Another study [42] compared depression and anxiety between PLP patients and other patients suffering from nonphantom neuropathic pain caused by trauma or surgery. Like another study mentioned above [36], this study used the HADS. This study found that amputees with PLP showed fewer symptoms of both depression and anxiety compared to patients with other types of neuropathic pain; however, residual limb pain was not accounted for. Fuchs et al. [43] reported on affective distress and pain-related interference as assessed by the West Haven-Yale Multidimensional Pain Inventory [44], a questionnaire assessing different dimensions of pain-related burden, in large samples of patients with chronic back pain and musculoskeletal pain compared to three amputee groups with (a) none but PLP, (b) non but residual limb pain, and (c) both PLP and residual limb pain. In line with the results from other studies [36, 40, 42], persons with either PLP or residual limb pain showed less affective distress and pain interference compared to chronic back pain or musculoskeletal pain patients. Concurrent PLP and residual limb pain was associated with higher burden compared to the groups that reported only one type of postamputation pain. However, even the amputees suffering from both pain types always showed less-intense burden than patients with chronic back pain or musculoskeletal pain. In a longitudinal study, Castillo et al. [45] assessed depression and pain in subjects with lower limb trauma at 3, 6, 12, and 24 months after injury. Pain predicted depression, but depression did not predict pain. It is important to note, however, that not all subjects in this study were amputees and that the study also did not make a distinction between PLP and other types of limb trauma-associated pain.

Taken together, these studies suggest that the association between pain and depression might be weaker in PLP than in other chronic pain conditions, including residual limb pain. In addition to a differentiation between specific pain types in amputees, it is important to consider the diagnostic tools and the composition of study samples.

### 2.3. Anxiety in Later Stages after Amputation

Anxiety is assumed to be both an etiological and a maintaining factor of chronic pain, especially as it is associated with avoidance of activity and perceived disability [46]. Similar to depression, anxiety symptoms are common in an early stage after amputation [27, 29], and anxiety levels decline in the first years after amputation [29, 33]. However, anxiety does not appear to be exclusively related to PLP shortly after amputation. Horgan and MacLachlan [33] stated that anxiety in acute amputees mainly relates to changes in body image, altered social role and social discomfort, and adaptation to a new identity. The preoccupation of amputees with topics relating to identity and social functioning (rather than pain) was also found in a qualitative study that explored amputees' experiences using a semistandardized interview [47]. Similar to what has been observed for depression, Whyte and Niven [31] also found that, in an early phase after amputation, anxiety was mainly correlated with somatic symptoms (e.g., insomnia) but not necessarily with pain.

There are only few studies on the association of anxiety and PLP after the immediate consequences of an amputation have subsided. The study by Desmond and MacLachlan [36] showed that compared to pain-free amputees, anxiety levels were higher in long-term amputees with chronic PLP or residual limb pain. However, those levels were still within the range of the healthy population. In their longitudinal study, Castillo et al. [45] showed that, in a late, chronic phase of pain following lower-extremity trauma, subsequent pain was predicted by anxiety but not by depression. However, as mentioned above, this study included amputees and nonamputees and did not differentiate between PLP and other types of pain.

## 3. The Role of Stress and Tension in PLP

Stress is thought to play a key role in the development and maintenance of chronic pain. As outlined in the diathesis-stress model of chronic pain [46, 48], various dysfunctional affective, cognitive, and behavioral responses mediate the relationship between stressors and pain symptoms in chronic pain patients. It has been proposed that similar to other types of chronic pain, stress can trigger pain episodes in PLP [24, 49]. Although some studies can be found, which will be discussed in the following paragraphs, there is generally less research on the role of stress in PLP compared to more common types of chronic pain (e.g., chronic back pain or headache [24]).

Sherman and colleagues conducted several studies on this topic and proposed that there are complex interactions between the central nervous system, sympathetic arousal, and characteristics of the residual limb [50]. For example, Sherman and Bruno [51] showed that the temperature of the residual limb compared to the intact limb is decreased in amputees with PLP, which has been related to decreased near-surface blood flow. The authors further showed a significant relationship between the extent of temperature differences and PLP quality such as burning, tingling, and throbbing. Discharges of peripheral input can be mediated by autonomic nervous system activity which could explain why situational (external) stressors and internal states (e.g., tension and anxiety) interact and trigger PLP episodes. In fact, Sherman et al. [52] demonstrated that there is a close temporal relationship between PLP and involuntary contractions of residual limb muscles: muscle activity bursts, which were recorded using surface electromyographic signals, preceded the experience of PLP. Involuntary contractions of residual limb muscles have been directly related to anxiety, tension, and stress [53]. This relationship is also supported by studies using relaxation-focused interventions to reduce PLP: In a review, Sherman [54] suggested that most treatments for PLP show success rates of 30% or below that might be merely due to placebo effects; only treatments reducing tension showed better effects and can be considered as successful treatments. Preliminary beneficial effects have also been shown for biofeedback. In a case study, total and enduring relief of PLP and an increasing temperature of the residual limb were observed following a residual limb EMG and temperature biofeedback training [55]. In another study, improvements in pain of at least 30% were observed in 6 out of 7 patients after six sessions of residual limb temperature biofeedback training [56]. However, this study lacked a control group, and better controlled studies on biofeedback in PLP are needed to evaluate its effectiveness.

Angrilli and Köster [57] experimentally investigated the effect of stress induction on amputees with and without PLP. Stress was induced in the amputees by means of three different induction methods: (a) having them deliver a free speech about memories of the amputation, (b) applying a cold pressor pain test, and (c) performing a mental arithmetic task. As measures of sympathetic stress responses, the authors recorded the heart rate and blood pressure levels. Amputees with PLP showed stronger psychophysiological stress reactions compared to amputees without PLP only in the free-speech task. This study thus suggests that amputees with PLP show enhanced stress responses, at least when the stressors are autobiographic and specific (e.g., reminders of their pain or amputation), similar to findings in chronic musculoskeletal pain patients [58]. Moreover, the results support the hypothesis that distressing pain memories (see below) play a role in PLP [59–61]. Heightened stress reactivity in amputees with PLP has also been demonstrated on a cortical level. Using electroencephalography, Larbig et al. [62] showed that PLP patients display increased cortical activity when presented with verbal pain-associated material.

The studies described above support the notion that PLP relates to both peripheral and central arousal and that PLP episodes might be influenced by emotional distress. Katz [63] proposed a model explaining the close connection between cognitive and affective processes and PLP. In amputees, the threshold for somatic sensations in the phantom limb might be lowered due to the loss of inhibitory control. The underlying mechanisms are proposed to be identical to those when healthy participants experience somatic sensations while affectively aroused. The reduced threshold for sympathetic activation might allow even events of much lower salience to trigger somatic sensations in the phantom limb, which further indicates that brain regions involved in cognitive and affective processes might contribute to a sympathetic-efferent/somatic-afferent cycle of activity. Interestingly, sympathetic arousal might also play a role in other chronic pain conditions and might further be related to maladaptive plasticity in the brain. For example, in patients suffering from complex regional pain syndrome (CRPS), sympathetic blocks have been shown to restore the organization in the primary somatosensory cortex [64]. CRPS is a condition characterized by ongoing pain perceived in upper or lower extremities, which shares similarities with PLP, as in terms of neuroplastic changes in the sensorimotor cortex [65, 66]. However, up to now, the contribution of sympathetic arousal to PLP remains largely unexplored.

How do these laboratory findings on the interaction between stress, peripheral or central arousal, and PLP relate to more naturalistic contexts and to daily life stressors? Giummarra et al. [67] developed a structured questionnaire on specific triggers of PLP episodes and investigated their frequency of occurrence in a sample of 264 amputees. Although behavioral triggers (e.g., trying to use the phantom) and stimulation of the residual limb (e.g., movement, touch, or pressure) were the most common trigger categories (50% and 37%, resp.), emotional triggers such as emotional distress, exhaustion, or thinking of the missing limb were still reported by 23% of the amputees. Moreover, 20% reported influences of the weather and another 11% reported about referred sensations originating from the intact limb. These results support the notion that PLP episodes often follow emotional distress and that residual limb activity interacts with PLP experience [52, 57]. However, these data are based on cross-sectional and subjective reports and may therefore be prone to memory biases or express implicit theories rather than actual events. Instead of using cross-sectional questionnaire data, Arena et al. [68] conducted a longitudinal study employing pain and stress diaries. Twenty-seven male amputees with PLP completed the diaries four times a day for six months. The authors performed a cross-lagged correlation analysis to detect relationships between stress and PLP over time. A significant relationship between stress and PLP was found in 74% of the amputees. The authors observed that, (a) in 63% of the sample, stress and pain covaried simultaneously, (b) in 44%, a change in pain preceded a change in stress, and (c) in 37%, a change in stress preceded a change in pain. This study thus supports the interpretation that even on the level of daily life stressors, there is a bidirectional relationship between PLP and stress. Taken together, findings from the laboratory and naturalistic observations are well in line with each other and support the role of stress in PLP.

Another reference to altered stress processing in PLP comes from studies investigating the importance of traumatization due to limb loss and initiated the developments of novel treatments of PLP [69]. Preliminary data indicate that a trauma-focused psychological treatment, aiming at traumatic amputation-related memories, successfully reduced PLP [70], probably due to the improvement of regulation of sympathetic arousal [71]. Consequently, an eye movement desensitization and reprocessing treatment, which has been applied in the treatment of posttraumatic stress disorder, showed promising effects in reducing PLP-associated suffering [72], although the specificity of this intervention needs to be clarified.

## 4. Cognitive Factors

Pain experience can be modulated by cognitive factors such as attention, memory, expectations, beliefs, appraisals, and (cognitive) coping strategies [24, 73, 74]. For many of these factors, little is known about how they specifically modulate PLP [18]. Although we do not know how attentional processes modulate PLP, some indirect indications exist. For example, hypnosis alters attentional processes and can be efficient in modulating both acute and chronic pain (for reviews, see [75, 76]). A reduction of PLP by hypnotherapy has been shown in a case study [77], although more studies are needed. Placebo effects, which are partly based on expectation [78], can also be used to alleviate neuropathic pain [79] and might potentially be effective in PLP although this has not been tested. Finally, the literature shows detrimental effects of catastrophizing on PLP, which are probably also mediated via attentional and expectation processes [80]. In the following sections, we will review relevant findings on memory, coping styles, and catastrophizing and their importance for PLP.

### 4.1. Memory for Pain

Several theories of chronic pain suggest that both declarative and nondeclarative memory processes contribute to the development and maintenance of chronic pain via neuroplastic changes in the nervous system [81–83]. One example for this process is central sensitization, referring to a hyperexcitability of the central nervous system and associated lowered pain thresholds [84]. In different groups with chronic pain, structural and functional reorganization within pain-processing brain areas has been shown, which might represent a neural correlate of pain memory [81, 85, 86].

For amputees in particular, memory processes have been discussed for both nonpainful phantom sensations and PLP. Katz and Melzack [61] and Katz [87] proposed a crucial role of *somatosensory memories* for both PLP and nonpainful phantom phenomena (e.g., phantom limb awareness or phantom sensations). Anderson-Barnes et al. [88] suggested that phantom sensations could be explained by *proprioceptive memories* and that they might become associated with pain perceived before the amputation by means of learning mechanisms. Due to tumor, vascular disease, or injury, an amputation is often preceded by pain in the affected limb. Katz and Melzack [61] suggested that pain prior to the amputation is encoded and can later be triggered, for example, by peripheral input stemming from the residual limb. This triggered preamputation pain is then experienced as PLP. Maladaptive plasticity that is positively correlated with PLP [65] can be seen as neural underpinning of pain memory in amputees. These processes might be more severe if chronic pain has already been present prior to amputation [81, 85, 86], potentially influencing the formation of a pain memory. The theory of a relationship between preamputation pain (that leaves behind a memory trace) and PLP is supported by retrospective reports that have shown similarities between memories referring to somatosensory perceptions in the affected limb in the phase before amputation and later phantom sensations [60, 61]. For example, in the study by Katz and Melzack [61], almost 60% of the amputees who reported having had pain before the amputation also reported that similar pain qualities continued or recurred later on in the phantom limb. However, reports about preamputation pain that were gathered retrospectively might be prone to memory bias. Prospective studies, on the contrary, give more valid information on the relationship between preamputation pain and PLP and showed that PLP during the first six months, but not long-term PLP, was predicted by pain before amputation [89, 90].

Finally, in line with the observation that chronic pain patients often complain about forgetfulness, impairments of both working memory and long-term memory have been discussed for chronic pain (for reviews, see [91–93]). Although empirical studies have used various methods and have found heterogeneous results when testing memory performance, reviews and meta-analyses indicate that chronic pain patients show poorer performance on memory tests [91–93]. In a recent review, Mazza et al. [91] have argued that poor performance in working and long-term memory tests of chronic pain patients might be related to impaired encoding and/or retrieval processes. In addition, this study also indicated a memory bias towards selectively remembering pain-related events. Whether processes of working memory or long-term memory are also associated with PLP has not yet been systematically studied.

### 4.2. Coping Strategies

The general term *pain coping* describes functional or dysfunctional styles to deal with pain after it has been attended to and interpreted (appraised) as being a threat [94, 95]. Pain coping can be divided into cognitive and behavioral strategies and also includes catastrophizing, which will be discussed in detail in the following section. Cognitive coping strategies comprise, for example, distraction from a sensation or reinterpretation of pain [96]. Examples for behavioral coping strategies are increasing or decreasing social or physical activity or seeking social or medical support [94, 96]. Hill [96] systematically described coping styles in amputees with PLP by having sixty male PLP patients complete the Coping Strategies Questionnaire [95]. Using principal component analysis, she found that the factor structure in PLP patients resembled the one originally discovered by Rosenstiel and Keefe [95] in a sample of chronic back pain patients. In the study by Hill [96], three main components were found—cognitive coping, helplessness, and pain denial—which explained about 20% of the variance in both PLP and psychological distress. In addition, most of the variance in these variables was accounted for by catastrophizing, representing a core facet of the helplessness factor. The author concluded that the repertoire of PLP sufferers contains only a limited amount of coping strategies that actually help to alleviate distress and pain and that successful coping rather means *not* to catastrophize. In a later study by Hill et al. [97], catastrophizing explained 26% of the variance in PLP, whereas other strategies only explained 3%. Interestingly, although male and female amputees do not differ in respect of PLP prevalence when the cause of amputation is controlled for, females report greater levels of pain interference [98]. This sex effect might contribute to differences in reported strategies to cope with PLP, with women showing significantly higher degrees of catastrophizing than males [98].

The conclusion that PLP patients use rather few coping strategies was confirmed by another study applying a different methodological approach. Whyte and Niven [99] had 89 amputees collect diary entries assessing PLP and coping strategies once per hour for one week. Instead of using standardized questions, coping strategies were captured using a free format. Analyses revealed that the amputees used a limited number of strategies that could be classified into distraction, relaxation, seeking support, exercise, manipulation of the residual limb, and drug or alcohol use. Importantly, none of the reported strategies was shown to be effective in reducing PLP. This study thus confirms that PLP sufferers tend to use less effective coping strategies.

### 4.3. Catastrophizing

Pain catastrophizing is a maladaptive cognitive coping style that is characterized by an exaggerated, negative orientation towards pain and anticipation of negative outcomes. There is evidence that catastrophizing predicts chronic pain and associated impairment [94, 100, 101]. Several studies found that catastrophizing was significantly positively correlated with the magnitude of PLP in amputees [80, 96, 97, 102–105]. Jensen et al. [103] found that between one and six months after amputation, the change in PLP and depressive symptoms could be predicted by catastrophizing and lack of social support and overtly solicitous responses from family members. In a later study [102], these results were replicated for a period of 12 and 24 months after amputation. Surprisingly, in these studies, more intense catastrophizing was associated with an *improvement* of PLP and depression symptomatology six and 24 months after amputation. These results, at first glance, seem contradictory; however, they might be explained by regression to the mean artefacts, caused by the strong correlation between PLP, depression, and catastrophizing at the first time point in the studies, and thus, there was little room left for the patients to further aggravate at the second time point. Hence, the lagged relationships in these two studies do not necessarily indicate that catastrophizing predicts improvement of PLP. Rather, the results highlight the importance of taking the initial magnitude of pain into account. Another longitudinal study supporting the aggravating effect of catastrophizing on PLP was performed by Richardson et al. [104]. These authors found that catastrophizing before the amputation significantly predicted PLP six months after the amputation, whereas pain before the amputation was only weakly related to later PLP.

Relationships between catastrophizing and PLP have been investigated in two studies by Vase et al. [80, 105]. In the first study [105], catastrophizing accounted for 35% of the variance in PLP after statistically controlling for depression and anxiety. In addition, catastrophizing correlated positively with wind-up-like pain, elicited by pinprick stimulation applied to the residual limb. Wind-up is a dynamical pain phenomenon which is usually assessed by repetitive application of moderately painful stimuli of equal intensity. Due to temporal summation processes, stimuli are perceived as increasingly painful. However, strong enhancement in perceived pain in this paradigm indicates exaggerated summation or sensitization processes, which have previously been reported for chronic pain patients [106, 107]. Vase et al. [105] suggested that catastrophizing and wind-up might interact and contribute to PLP. In the second study [80], the authors used electroencephalographic recordings of cortical responses to noxious and nonnoxious stimuli presented at the amputees' affected and nonaffected limbs. For stimuli presented on the affected side, there was a significant correlation between catastrophizing and the power at the N/P135 dipole, suggesting an origin of cortical activity in the area of the secondary somatosensory cortex. The authors concluded that this finding might be explained by the fact that catastrophizing implies hypervigilant attention to noxious and nonnoxious stimuli. Another study showed that catastrophizing is not only related to PLP but also to disability as it predicted physical and psychosocial disability in amputees [108].

## 5. Changes in Body Perception and Their Relationship to PLP

The changes in physical integrity after amputation are accompanied by alterations in cortical body representation and body perception. Moseley et al. [109] introduced the body matrix concept, describing a widely distributed frontoparietal brain network involved in processing a combined representation of body and space for maintaining homeostatic control and enabling protective behavior. Amputation-induced changes in this network and their behavioral and perceptual consequences have been repeatedly studied in the past (e.g., [110–113]).

Previous studies have shown that brain changes in amputees with PLP compared to amputees without PLP are pronounced and that PLP intensity is positively correlated with shifts in sensorimotor body representation (e.g., [65, 114]). These reorganization processes appear to be mirrored in altered body perception. For example, reorganization in the primary sensory cortex is not only correlated with PLP but also with the experience of a telescopic distortion (e.g., [115]), that is, the perception that the phantom limb shortens or, in extreme cases, retracts into the residual limb over time. Other cognitive alterations were observed for functions involving spatial transformations of body parts that appear to involve posterior and superior parietal regions and intraparietal regions (cf. [116]). Thus, amputees with PLP perform worse when they have to mentally rotate their limbs (e.g., [117]), and a negative association between performance in mental limb rotation and the frequency of PLP has been shown [118]. This suggests that phantom sensations in general [119] and PLP in particular are associated with impairments in the body schema, describing a flexible central representation of body posture that is needed for action. Since mental rotation of body parts involves motor imagery processes [120], and since the associated neural mechanisms are different in amputees with PLP compared to amputees without PLP [121], these results highlight the impaired mentalization of motor execution specifically for painful phantom limbs. Consequently, it has been suggested that the central body representation itself should be a target of therapeutic approaches [122], and thus, novel treatments aim at normalizing the underlying processes [123–130]. Interestingly, the amputees' body perception might be important for the effectiveness of these treatments [6, 127].

The PLP-specific alterations in body representation also have consequences for higher-order emotional and cognitive processes. In a nationwide study, Bekrater-Bodmann et al. [4] surveyed a large cohort of amputees for the presence of residual limb pain and PLP and additionally for their body-related dream content. Specifically, they assessed the proportion with which the amputees dreamed of themselves as being impaired (i.e., amputated) versus having an intact (i.e., nonamputated) body. The majority of amputees recalled their body appearance in dreams as intact, even decades after the amputation. PLP correlated positively with the recall of an impaired body in dreams, indicating that suffering in the awake state influences body representation in dreams. This relationship was rather unspecific because for residual limb pain, the same relationship was found. However, although dream content is rather difficult to interpret, these results suggest that the impaired physical body has a higher salience in postamputation pain sufferers than in pain-free amputees. In line with this notion, there is new evidence that PLP is related to implicit attitudes towards amputated bodies in general. By using an lmplicit Association Test, Macauda et al. [131] showed that amputees implicitly prefer intact bodies. In fact, amputees did not differ from a nonamputated control group. Interestingly, PLP intensity was significantly and positively correlated with the preference for intact bodies. It remains open how these findings are related to those from an earlier study [132] in which amputees were instructed to draw their body images and in which amputees suffering from PLP drew their bodies as intact more often than amputees without PLP. To which extent this is mediated by emotional factors remains to be determined. Prospective studies need to carefully consider and examine the complex interplay between PLP, body-related higher-order cognitions, and emotional processing.

## 6. Summary and Conclusions

In this review article, we summarized and discussed the research on emotional factors, stress, cognition, and body perception in PLP. Most research on emotional factors focused on depression and anxiety. Rates of depression and anxiety are high in early stages after amputation; however, both conditions are probably not (or only weakly) associated with the occurrence of PLP but rather with problems related to the adaption to the new situation. In later stages, depression and anxiety might contribute to PLP, but these associations appear to be weaker than in other types of chronic pain. Up to now, it remains an open question why depression and anxiety might be less relevant in PLP than in other chronic pain states. One reason, which has already been suggested by Desmond and MacLachlan [36], might be that PLP less strongly interferes with everyday activity—and compared to residual limb pain even with prosthesis use—than other chronic pain syndromes. Another reason might be that, in the case of chronic back pain, emotional and cognitive variables might play a more important etiological role. For example, the fear-avoidance model of chronic pain [14] states that fear and avoidance of movement are acquired through a fear-conditioning process that might favor chronicity. Over time, avoidance of movement leads to disability and recurrent pain, which strengthens the learned association and leads to a vicious circle consisting of pain, disability, and fear. The model therefore suggests that fear conditionability might be a risk factor for chronic pain that is acquired in such a way. That chronic back pain patients show enhanced fear conditioning has been shown in several studies (e.g., [133]). The role of anxiety and negative affect in the acquisition of movement-related fear has also been shown experimentally [134, 135]. Another series of neuroimaging studies further suggest that emotional learning processes predict the transition from the acute phase to the chronic phase in back pain (for a review, see [136]). Whether these conditioning and emotional learning mechanisms are also important in PLP needs to be addressed in future studies.

Concerning stress, we showed that research, on the whole, suggests that there are bidirectional relationships between stress and PLP, probably due to interactions of the central and peripheral nervous systems. The bidirectional relationships between stress and PLP have been demonstrated both in experimental studies and more naturalistic settings. Given the relatively clear picture that can be drawn from this line of research, the utility of altering central and peripheral stress responses in clinical interventions should be explored. More recent studies also suggest that therapeutic interventions used in traumatization can be useful to treat PLP.

Little is known about the role of cognitive factors such as attention/distraction or expectation, which are commonly discussed as mediators of the pain experience. Memory is a well-represented topic in the PLP literature. However, it has not been clearly delineated which declarative and nondeclarative components are relevant and how they relate to neuroplasticity. Research on the role of catastrophizing in PLP shows that this coping style is positively associated with PLP presence, and longitudinal studies further indicate that catastrophizing aggravates PLP. Apart from avoiding catastrophizing, little is known about effective coping with PLP. Finally, we briefly discussed the role of body representation and body perception in PLP on which there have been many studies in the recent years and which are often perceived as the most promising targets for treating PLP (for reviews, see [137–140]). We also found evidence that cortical body representations might interact with psychological variables, but that until now, only few studies have focused on connections between these topics.

Compared to other types of pain, there has been little research on the role of psychological variables in PLP. whether psychological variables are less important for PLP or whether they have merely been neglected needs to be determined in future studies that take emotional, motivational, perceptual, and cognitive variables into account.
